# Human Consciousness: Where Is It From and What Is It for

**DOI:** 10.3389/fpsyg.2018.00567

**Published:** 2018-04-23

**Authors:** Boris Kotchoubey

**Affiliations:** Institute of Medical Psychology and Behavioral Neurobiology, University of Tübingen, Tübingen, Germany

**Keywords:** awareness, communication, embodiment, objectivity, play, tool use, virtual reality

## Abstract

Consciousness is not a process in the brain but a kind of behavior that, of course, is controlled by the brain like any other behavior. Human consciousness emerges on the interface between three components of animal behavior: communication, play, and the use of tools. These three components interact on the basis of anticipatory behavioral control, which is common for all complex forms of animal life. All three do not exclusively distinguish our close relatives, i.e., primates, but are broadly presented among various species of mammals, birds, and even cephalopods; however, their particular combination in humans is unique. The interaction between communication and play yields symbolic games, most importantly language; the interaction between symbols and tools results in human praxis. Taken together, this gives rise to a mechanism that allows a creature, instead of performing controlling actions overtly, to play forward the corresponding behavioral options in a “second reality” of objectively (by means of tools) grounded symbolic systems. The theory possesses the following properties: (1) It is anti-reductionist and anti-eliminativist, and yet, human consciousness is considered as a purely natural (biological) phenomenon. (2) It avoids epiphenomenalism and indicates in which conditions human consciousness has evolutionary advantages, and in which it may even be disadvantageous. (3) It allows to easily explain the most typical features of consciousness, such as objectivity, seriality and limited resources, the relationship between consciousness and explicit memory, the feeling of conscious agency, etc.

*The buttock, however, in man, is different from all animals whatsoever. What goes by that name, in other creatures, is only the upper part of the thigh, and by no means similar*.

George Louis Leclerc de Buffon (1792, pp. 80–81)

*Why do people think? Why do they calculate the thickness of walls of a boiler and do not let the chance determine it? Can a calculated boiler never explode? Of course, it can. We think about actions before we perform them. We make representations of them, but why? We expect and act according the expectancy;… Expectancy [is] a preparatory action. It outstretches its arms like a ball player, directs its hands to catch the ball. And the expectancy of a ball player is just that he prepares arms and hands and looks at the ball*.

Ludwig Wittgenstein (1996, pp. 109, 139)

The two epigraphs already give partial, but essential, answers to the questions in the title. Where human consciousness is from? In a large extent, it is from the exceptionally extensive tool use, which would be impossible without the erectness supported by the exclusively strong gluteal muscles. What is its function? As indicated by Wittgenstein, it is a set of simulated anticipations.

Notwithstanding substantial differences, most contemporary theories of consciousness (e.g., Dennett, 1991; Damasio, 1999; Edelman and Tononi, 2000; Koch, 2004; Maia and Cleeremans, 2005) regard it as a kind of information *processing*. The present paper, in contrast, regards it as a kind of *behavior*. Behavior is a biological adjustment by means of movements and all kinds of movement-related physiological activity (see Keijzer, 2005, for general principles of the modern theoretical analysis of behavior). Of course, the brain plays a critical role in the control of behavior. Complex forms of behavior (including consciousness) necessarily require, and become possible due to, the complexity of the controlling brain. But there is no isomorphism between a controlling system and a controlled system.

The paper is not about neural correlates of consciousness (NCC). I just do not find the problem of NCC very interesting for several reasons, the simplest of which is: correlation is not causation. Further, it is not about the so called hard problem of consciousness (Chalmers, 1996). The starting point of the present considerations is actively behaving organisms able to various forms of learning (mainly, associative learning). I assume that thus behaving organisms already possess something that can be called “core consciousness” (Damasio, 1999).

By the term “human awareness”, I mean, in accord with most philosophers of mind (e.g., Searle, 2000; Beckermann, 2005), the ability to experience one's own “internal states” as intentional states (Brentano, 1982), i.e., internal states that are “about” some external objects. This term does *not* imply that all components of this “human awareness” are uniquely human or that this kind of consciousness cannot be found in any nonhuman animal.

Several aspects of the presented model are already described in other published or submitted texts. In such cases only a very brief summary will be given here, and the reader will be referred to further papers. I understand that this way of presentation is highly inconvenient, but the space in open access journals is too valuable to afford the luxury of repetition.

The structure is as follows. First, I describe *precursors* and the three main *behavioral components* giving rise to human consciousness. Second, I describe a “*central block”* of human consciousness built on the interface between these three components (see Figure 1). This part is the least original for the simple reason that description of human consciousness has been undertaken by numerous thinkers from St. Augustin to modern cognitive scientists, and a completely novel description is hardly possible. However, this section is necessary to show how the extant descriptions follow from the three components displayed in the first section, and to put it apart of alternative descriptions.

**Figure 1:**
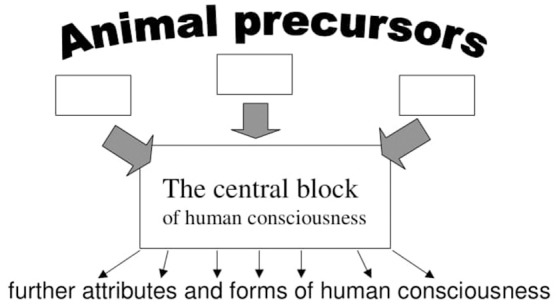
The direction of the explanation starts from relatively well-understood animal precursors to the central block emerging immediately from these precursors. Having understood this block, understanding of further attributes and forms of human consciousness can be hoped.

Third, we describe several most peculiar *features* of human consciousness to show how easily they are deduced from the presented model. Finally, we briefly regard the relationships between this model and some other, similar or remote theories of consciousness. Again, I ask for understanding that, for the above-mentioned space reasons the two latter points cannot be discussed in full in the present text; this discussion remains a topic of a separate analysis.

## Precursors

### Anticipation, core consciousness, and preconditioning

The organism/environment system is to be kept in a state of extreme energetic nonequilibrium (Schrödinger, 1944). Life, therefore, is the continuous battle against the Second Law of Thermodynamics. All organisms' needs, from the need of a paramecium in food to the need of a composer to write a symphony, can be subsumed as a need in negentropy, in making order out of energetic death.

To maintain the highly improbable energetic state, organisms interact with their environment in a continuous process of anticipatory regulations. “Regulations” means that environmental disturbances are steadily compensated to make possible the “necessary condition for free an independent life”: the stability of the internal milieu (Bernard, 1865). “Anticipatory” means that physiological regulations at the moment *t* are such as to compensate the disturbances at the moment *t*+*1*. This is particularly true for moving animals. The more mobile is an organism, the more distant is the organism's environment in terms of space, the more ahead of the present point it must be in terms of time.

However, the future cannot be known for sure. Anticipatory processes can therefore be regarded as hypotheses built by the organism about the future state of its environment. All biological adaptations are merely “assumptions to be tested by the selection” (Eibl-Eibesfeldt, 1997, p. 28). Behavioral adaptations, however, are even more hypothetical than, say, morphological adaptations, because they can be tested immediately after an action rather than later in the life. Behavior is principally anticipatory, i.e., based on prediction and correction of upcoming environmental disturbances (Grossberg, 1982; Rosen, 1985; Friston, 2012).

Several authors including, e.g., Bickhard (2005; Bickhard et al., 1989) and Jordan (1998, 2000; Jordan and Ghin, 2006) indicate that anticipatory interactions give rise to core consciousness. The emergence of primary elements of consciousness (“the hard problem”) is beyond the topic of the present article. Of course, it is difficult to know and even more difficult to describe “what it is like” to have *only* core consciousness. Any description of a qualium (if there is such a thing), requires a term (e.g., “redness”: Humphrey, 2006) which belongs to much higher levels of consciousness than the described phenomenon itself. In any case, our object here is not the emergence of such simple forms of consciousness but a very long way from them to that Cartesian cogito that we usually conceive of as our human awareness.

Anokhin (1974) demonstrated that all mechanisms of conditioning, including both Pavlovian (classical) and Skinnerian (operant) processes, can be considered as anticipatory phenomena. However, an important modification of the classical conditioning procedure is particularly interesting for the following development. In this modification, called preconditioning (Brodgen, 1939), subjects are presented a contingent pair of neutral stimuli (S1–S2; e.g., light and tone), none of them having biological significance. Not surprisingly, their combination does not yield any observable effect. Subsequently, S2 is paired with a typical unconditional stimulus (UCS, e.g., food), leading to a classical conditioned response (CR, salivation). After this, the first neutral stimulus (S1) is presented. Surprisingly, it also elicits a CR, although it has never been combined with the UCS.

The fact that stimuli having no reinforcing value can nevertheless affect behavior was a big challenge for behaviorist theory (see Seidel, 1959, for review). In fact, preconditioning implies two different achievements. First, a new dimension of *time* is opened. The association between S1 and S2 must be retained in memory until S2 is combined with UCS. Even more importantly, the animal's brain should have sufficient complexity to make use of *contingency of nonsignificant events*. Obviously the animal could not know that one of the stimuli would acquire a meaning in the subsequent conditioning. Therefore, it must be in possession of free resources to record some statistical regularities in the “environmental noise,” whose meaning is momentarily zero. The only purpose of this enormous vast of resources is that some of these presently meaningless combinations may (perhaps) become meaningful in future.

Preconditioning is widely presented among different vertebrate species even in a very young age (e.g., Talk et al., 2002; Barr et al., 2003). Recent data indicate the possibility of sensory preconditioning in bees (Müller et al., 2000) and fruit flies (Brembs, 2002).

Up to this point, the animal lives in the world of its needs, among those relevant events that either satisfy these needs or counteract the satisfaction. This *Lebenswelt* is determined by the genetic design of the animal and continuously extended by learning: new features are revealed as soon as they are associated with genetically hard-wired biologically significant features. An organism is engaged only in those interactions it is designed for (Maturana, 1998). This simple law is first broken by preconditioning: the animal learns to pay attention to *meaningless* events. It overcomes immediate satisfaction of its needs and begins to “save information” with the hope that sometime it may become useful.

### Play

Preconditioning is association of two external elements (“stimuli”) having no immediate consequence. Likewise, play is association of *organism' own actions* and external events, also having no immediate consequence. This definition leads us to distinguish between the immediate and the remote gains of an activity. On the one hand, play is “for fun” (otherwise it should not be called so), on the other hand, it can have a serious function. This vast of energy could not be so ubiquitous in nature if it is not compensated by some important gain. This contradiction between the immediate uselessness and the remote usefulness of play is probably the central point of all ethological, culturological and philosophical discussions to this issue.

Frequently, play appears to copy a serious activity (Burghardt, 2005). Play superficially imitates hunting, sex, aggression, but it is not what it seems. However, the imitative character is not obligatory. When parrots or monkeys just hang on branches and swing without any purpose, most human observers would find that they are playing, although such hanging and swinging does not appear to resemble any serious activity.

Although it is sometimes very difficult to decide whether a particular activity is a play, most people agree that many mammals and birds play (Burghardt, 2005). Playing octopuses have also been described (Kuba et al., 2006). Some groups likes predators, primates, sea mammals, and solipeds play distinctly more than others. Further, in all nonhuman species youngsters play considerably longer time (by a factor between 10 and 100) than adults. Already Lorenz (1971) noted that youngsters are more ready to learn nonsense.

An important feature of play is security. In play, skills can be exercised without a risk to fail. When a predator fails in hunting, it can die of hunger. (I mean wild animals, of course, not the pets cared for by old ladies.) A youngster which fails in hunting play will be alimented by its parents. Play, therefore, introduces something that can be called “second reality” (Vygotsky, 1978). In this reality the life is going on as if it is the “primary reality,” but with the nice difference that whenever I don't like what happens, I simply stop the process and go out, or start it anew. This makes play suspiciously like consciousness. Coaches of athletes are aware of this similarity, thus they combine training without real competition (which is play, as competition is reality) with mental imagery (which is a typical phenomenon of human awareness).

Play is only relatively secure, however. A hunting play takes place in the same real world as the real hunt. Accordingly, although the dualism between play and reality is presented in philosophical thinking (Huizinga, 1950), it is not as strong and troubling as the dualism between mind and matter. Although the result of the playing activity is not of vital importance, the circumstances are real, and obstacles are material, thus the animal can be seriously injured. Remember how many soldiers in the armies die, not in a war, but in training.

### Tools

Play is the first important consequence of the ability to learn without reinforcement. The second consequence is the use of tools. The role of tools in creating the world of objects, and of the very distinction between objective and subjective, is analyzed in Kotchoubey (2014). A tool is a neutral component of the environment used to operate with other components which, in turn, are related to the animal's survival. For example, a stick is neither eatable nor dangerous; but it can be used to reach fruits or to fight enemies. Manipulation with a tool cannot be successful from the very first trial. A stick is eventually manipulated “just for fun,” and then, suddenly, it turns out to be useful. Thus no animals unable to play can use tools.

Remember that animals do not worry about the world “as such” (von Uexküll, 1970). They know (i.e., can efficiently deal with: Maturana, 1998) those elements and features of the world which are related to the animal's needs. This can be illustrated by the following scheme:

[Me] < = = > [something in the world as related to me]

(note that “me” is presented on both members of the scheme!)

Using a tool, an animal gets knowledge about a new kind of qualities: qualities which relate an element of its environment, not to the animal itself, but to other elements of the environment. For example, a stick used by apes for reaching bananas should possess the feature of hardness:

[Me] < —— > [a component of the world] < - - - - > [another component related to me]

The dashed line < - - - - > indicates a newly acquired relation between two components of the outer world. From this moment I possess a bit of “objective” knowledge, in the sense that I know about some aspects of the world which are unrelated to my own being in this world. Bananas are eatable only as long as there is me who can eat them. Sticks, to the contrary, will remain hard independently of my existence. My knowledge of this hardness is, therefore, objective knowledge, and the corresponding aspect of my environment (i.e., the stick), is now an object. Also the solid line < —— > represents a new kind of relations: my ability to operate with tools and to choose means appropriate to my ends.

The objectivity of the obtained knowledge is, however, limited by the fact that the object in question remains related to me in two aspects: as my tool (something in my hand) and as an immediate mean to my goal (the banana). The object is “compressed” between me and me; it does not have an open end. Some animals, however, can make one step more combining two tools, as chimpanzees in famous experiments of Köhler (1926). This can be depicted as:

[Me] < —— > [object 1] < ~~~~~ > [object 2] < - - - - > [my goal]

The twisting line < ~~~~~ > between two objects stands for a relation from which I am completely factored out. For a long time after these experiments, it was believed that using higher-order tools is limited to most intelligent primates: humans and some apes. Now we know that using and making tools is widely presented among numerous animals including birds. Some parrots (like kea) particularly dexterous in tool usage are also particularly playful (Huber and Gajdon, 2006). Crows, ravens and finches use twigs, petioles, leaf stems and wire in both experimental and natural environment (Hunt et al., 2006; Watanabe and Huber, 2006). They flexibly adjust the length of their tools to the perceived distance from the food, prepare leaf stems for more convenience and use stones to bend wire or make hooks (Weir et al., 2002). They can use a short stick to reach a long stick to reach the food with the latter (Taylor et al., 2007). Chimpanzees, keas, and New Caledonian crows know something about the *objective world*.

### Communication and symbols

Another important precursor that added to play and tool usage arises from communication. Communication can be defined as a behavior whose *main effect* is changing the behavior of another animal of the same or different species. A similar definition regards communication as the whole of biological effects *selected for* their influence on other animals of the same or different species. Any directed effects on another's behavior require transmission of signals between animals, and signals have a property to stand for something different from the signals themselves. According to the classical tripartition of Peirce, signals can be symbolic, iconic, and indicative (Buchler, 1955). Most animal communication signals belong to the category of indices. Indices are signs causally related to their reference; classical examples are medical symptoms indicating a particular disease. Fever and headache do not just “mean,” or refer to, influenza, they are caused by the viral infect. Likewise, a danger scream is not arbitrary (like the word “danger”) but physiologically related to the corresponding emotions of fear and anxiety. Also human exclamations such as “Ah!” and “Wow” are causally related to particular emotions and not products of agreement. They are parts of the organism's state and not just signs of this state (Kotchoubey, 2005a,b).

But a danger scream, though physically related to the state of fear, is not physically related to the cause of this state, e.g., the appearance of a predator. Different animal species use completely different signals to signalize the same thing. The signals can be flexibly adjusted to the kind of danger (Donaldson et al., 2007), as vervet monkeys, for example, produce different alarm signals for leopards, eagles, and snakes (Cheney and Seyfarth, 1990). Slowly, the progressive differentiation of signals can yield the ability to integrate them in new ways: a combination of two alarm signals may not be an alarm signal at all, but acquires a different meaning (Zuberbühler, 2002).

Although both animal communication and human speech are mainly vocal, most authors agree that there is probably no continuity between animal cries as indicative signs and human language constituted mainly from symbolic signs (Ackermann et al., 2014); rather, the decisive change was made within gestural communication (Arbib and Rizzolatti, 1996; Corballis, 2002; Arbib and Bota, 2003). Our closest ancestors in the animal world were not the best in vocal communication. Gestures, i.e., signs made by extremities independently of locomotion, exist, however, only in humans and apes and have not been found in any other animal including monkeys (Pollick and de Waal, 2007). Although animal cries also can be modified by context (Flack and de Waal, 2007), gestures are much more context-dependent (Cartmill and Byrne, 2007; Pika and Mitani, 2007), more flexible and thus their causal links to the underlying physiological states are much weaker as compared with vocalizations (Pollick and de Waal, 2007). Gestures thus paved the way for that separation between the signified and the signifying, which is so characteristic for true human language (e.g., Herrmann et al., 2006).

An interaction of communication signals with another factor mentioned above—namely, play—yields an emergent quality of playing *games*. Already Huizinga (1950) noted that any full-blown language is only possible on the basis of play as its precondition in contrast to animal communication that may not be related to play. Even relative independence of signs on their references can be used by an organism possessing the ability to play, which can now play with these signs, recombine them in deliberate combinations. The way is now open for conditional rules and systems of rules which might appear as strict as natural laws but, in contrast to those latter, they are not physically necessary. The notion of language as a symbolic game is frequently attributed to Wittgenstein (e.g., Hacker, 1988, 1990), who repeatedly compared language with chess (Wittgenstein, 2001).

Like the combination of symbols and play produces to the ability to play games, the mutual fertilization of symbols and tools brings about a new set of abilities to put *remote goals* and to relate them to actual sensorimotor interactions. The former complex gives rise to language, the most universal symbolic game all people play; and the latter gives rise to praxis (Frey, 2008). *Language and praxis* are two domains of abilities very specific for humans. Both require a famously unique feature of the human brain, i.e., its strong hemispheric asymmetry; both aphasias and apraxias result almost exclusively from lesions to the left hemisphere. The exact evolutionary process of this interaction is not completely clear. Gibson (1993) proposed that the use of tools may have caused the transition from mainly vocal to primarily gestural communication. On the other hand, Frey's (2008) review indicates that, more plausibly, tool use and gestures first developed as two independent systems and later interacted to produce human practical abilities.

## The second reality of consciousness

Now we have all the necessary components together. The organism, which already at the beginning could learn through interactions with its environment, has acquired several additional facilities. Being able to manipulate a hierarchy of tools, it can now discriminate objective features of the world, that is, not only the features relating something to the organism but also features relating things to each other. The organism further possesses a developed system of signs and can distinguish (a) between a sign and an internal state, which the sign signifies; as well as (b) between the sign and an external object which the sign refers to. It can play with these signs, recombine them and construct complex systems of arbitrary rules for symbolic games.

Taken separately, communication, play and tool usage are broadly presented in nonhuman animals. None of these abilities can be said to coincide or strongly correlate with thinking or culture. None is limited to one particular group of our human ancestry, e.g., only to primates or mammals. None is related to a particular type of nervous system (Edelman et al., 2005); in fact, none can be said “a cortical function” because these behaviors are observed in birds (whose telencephalon is only a remote homolog to the mammalian cortex), and sometimes in insects and cephalopods, with their completely different neural morphology.

**Figure 2:**
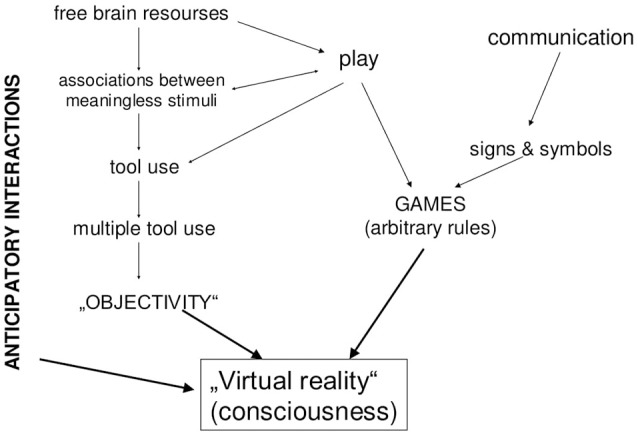
Main sources of human consciousness.

But the *combination* of communicative skills, play and tool use makes a qualitative jump. A being that possesses all these features can do the following: in a difficult situation, in which several behavioral alternatives are possible, it can experience something like “playing forward” (re. Play) each alternative, using symbols (re. Communication) referred to objective knowledge (re. Tools) about its environment. This process, i.e., internalized playing behavioral options, which takes into account objective features of the elements of environment and employs symbolic means, is nothing else but human conscious thinking (Figure 2).

### The virtual space

The model of human consciousness we use here is a virtual reality (VR) metaphor (Baars, 1998). The main block of human consciousness is anticipatory behavior in a secure “virtual” space of symbolic relationships, in which this behavior does not have any overt consequences. Behavior is thus anticipated by playing it forward in the realm of objectively grounded symbols.

Unfortunately, VR metaphor has also been used in a meaning completely different from the present one. Revonsuo (1995) regarded all experience as VR created by our brains and having nothing in common with “something there.” “The neural mechanisms bringing about any sort of sentience are buried inside our skulls and thus cannot reach out from there—the non-virtual world outside the skull is … black and imperceptible” (p. 13 of the electronic source). He proposed a horrifying description of reality as “The Black Planet” in which we “cannot see anything, hear anything, feel anything.” We can only construct a virtual world, but the real world outside will forever remain “silent and dark.”

In a similar vein, Metzinger (2003) developed an elaborated model of consciousness largely based on the Revonsuo's (1995) VR metaphor:

“Neither the object component nor the physical body carrying the human brain has to exist, in principle, to phenomenally experience yourself as being related to certain external or virtual objects. … Any physical structure functionally isomorphic to the minimally sufficient neural correlate of the overall-reality model … will realize first-person phenomenology. A brain in a vat, of course, could—if approximately stimulated—activate the conscious experience of being a self in attending to the color of the book in its hands, in currently understanding the semantic contents of the sentences being read, or in selecting a particular, phenomenally simulated action … the discovery of the correlates … could never help us decide is the question of how the brain in a vat could ever know that it is in this situation—or how you could know that you are now not this brain.” (Metzinger, 2003, p. 415).

This *solipsist* VR metaphor should be strongly distinguished from the *instrumentalist VR* metaphor presented here. By the term “virtual” I mean “artificial” or “second-order,” but *not* “unreal,” “illusory,” or “apparent.” Play hunting is an artificial activity as compared with real hunting, but it is not an illusion of hunting. For example, in Pavlovian second-order conditioning an animal responds to a tone paired with a light after the light has been paired with food. The animal salivates to the tone although it has never been paired immediately with food, but only with the light. Pavlov might have called the light in such experiments a “virtual reinforcer” if the term “virtual” was current those days. But he would have been greatly surprised if a philosopher explained him that what he means is just an “appearance” of reinforcement, that there is no such thing as food in the animal's world, and that there is no world whatsoever around the animal.

Speaking that human awareness “models” or “simulates” reality, we should understand the meaning of the corresponding terms (Northoff, 2013; van Hemmen, 2014). Every physical or mathematical model building necessarily presumes some pre-knowledge about the process to-be modeled. To build a model of something we must already have an idea of what this thing is and what properties it has. Model building is always a way of testing the hypotheses formulated before we have started to model. Who states that our conscious awareness (or the brain as its organ) models the world, presumes that the world already exists independently of our awareness (Kotchoubey et al., 2016).

The obvious advantage of virtual behavior is the ability of secure learning. The price for learning can be an error and the price for an error can be a failure, an injury or even death. Thus the optimum would be an area in which we may commit errors without being punished for them but, nevertheless, learning from them. This virtual area is consciousness. In words of Karl Popper, instead of dying as a result of our errors, we can let our hypotheses die on our site (Popper, 1963).

On the other hand, the adaptive action is postponed (vast of time), and the resources are consumed for virtual activity having no immediate effect (vast of energy). Therefore, conscious behavior is worth only in particularly complex situations in which its gains overweigh its losses: (1) when there are several behavioral options whose consequences are unclear, or whose chances appear similar; (2) when a risk of negative consequences of a wrong action is high, i.e., when the situation is dangerous but the danger is not immediate. Then, the disadvantage of the delay is overbalanced by the advantage of withholding an erroneous action.

Therefore, speaking about “human awareness,” we do not mean that humans are in this state all the time. This would be a catastrophic vast of resources that the Mother Nature never could afford. Again, this does not mean that otherwise we behave “unconsciously.” We just interact with our environment, we are *engaged* in our world. As Heidegger (1963) showed, this engagement is beyond the dichotomy of conscious vs. unconscious. We live in this engagement and experience it, and in this sense, we are conscious (Kadar and Effken, 1994), but we do not consciously think. We are just there (Heidegger, 1963; Clark, 1997).

This “ordinary state” of human existence can, of course, be compared with animal consciousness. We are thrown in our *Lebenswelt* like animals are thrown in theirs. However, our world is not theirs. The world in which we are engaged even without exerting conscious control is a social and instrumental world, i.e., it is already built up of those elements (tools, communication, symbolic games) which gave rise our conscious behavior. Many important differences between humans and apes result from the differences in their environment (Boesch, 2006). Most of our activity is automatic, like in animals, but these automatisms differ in both design and content (e.g., Bargh and Chartrand, 1999). Our existential reality is cultural, and so are our automatisms. Whitehead, who claimed that “civilization advances by extending the number of operations which we can perform without thinking” (Whitehead, 1911, p. 61), illustrated this idea not with automatic catching or grasping, but with automatized operations of experienced mathematicians over algebraic symbols. This automatization is hardly attainable even for very clever apes.

### Objections

Three arguments, which are frequently put forward to defend the solipsist variety of VR, can be regarded as objections against the present instrumentalist version: illusions, dreams, and paralysis. The illusion argument reads that consciousness cannot be deduced from interactions between organism and reality because in some mental states (e.g., illusions or delusions) we experience something different from reality. The argument is, first, inconsistent because who says that illusory perception is different from reality implies that he knows reality. Second, the argument confuses adaptive and maladaptive consciousness. Illusions in humans adapted to their environment occur only in very specific conditions on the background of millions of cases of veridical perception. Those whose mental life is prevailed by illusions, in contrast, are usually unable to survive without modern medicine and social support. The argument misses, therefore, the adaptive function of awareness.

The use of dreams in this context is equally misleading. The neural basis of dreams, the REM sleep, is completely different from the waking state in terms of physiological, humoral, neurochemical and behavioral components (Hobson and Pace-Schott, 2002; Pace-Schott and Hobson, 2002). Accordingly, dream experience has a number of formal (regardless of dream content) properties qualitatively different from those of our ordinary experience (e.g., Hobson, 1988; Stickgold et al., 2009). Lisiewski (2006) distinguishes between a “strong” and “weak” VR in the set-theoretical sense. “Strong” VR keeps constraints close to the constraints in the real world as it is experienced in simple forms of sentience. Examples are typical existing VR programs. In a “weak” VR, in contrast, all constraints are removed, e.g., one can fly, be simultaneously two persons, observe oneself from the side, etc. Dream consciousness, like science fiction stories, belongs to the latter category. That is why in dreams the muscle tone is nil, a finding predicted by Freud half a century before this fact was empirically proven (Freud, 1953). Freud's reason was that when reality constraints are removed, subjects must not have a possibility to actively behave. Therefore, dreams cannot be used as a model of conscious experience because the very essence of dream states is the blockade of the interaction between the organism and its environment.

The paralysis arguments indicates that humans extremely paralyzed for a long time (locked-in syndrome: LiS) and, therefore, lacking all overt behavior, nevertheless retain consciousness and can demonstrate very high intellectual functions (e.g., Kotchoubey et al., 2003; Birbaumer et al., 2008). However, all described cases of LiS (mainly a result of a brainstem stroke or of severe neurodegenerative diseases: for a review see Kotchoubey and Lotze, 2013) concern adult individuals, usually older than 30. All of them possess many years of experience of active behavior. From a philosophical viewpoint it might be intriguing whether consciousness would develop in a child with an inborn LiS, but from the ethical viewpoint we should be glad that no such case is known.

In most LiS patients, at least some movements (usually, vertical eye movements) still remain intact. Due to the progress of medicine and assistive technology, now many locked-in patients can use the remaining movements for communication and describe their experience in the acute period of the disease (e.g., Bauby, 1997; Pantke, 2017). These patients' reports show that the patients, albeit conscious and in possession of higher cognitive abilities, do not have “experiences as usual” as was previously believed. Rather, LiS is related to subtle disorders of conscious perception and attention that cannot be explained by the lesion of motor pathways but probably result from the dropout of motor feedback (Kübler and Kotchoubey, 2007; Kotchoubey and Lotze, 2013).

Using a Brain-Computer-Interface (BCI), cognitive abilities of LiS and other severely paralyzed patients can be checked independently of their motor impairment (e.g., Birbaumer et al., 2008). The analysis of the corresponding data shows that the ability to learn in patients, who possess minimal remaining movements (maybe only one), is only slightly, if at all, impaired in comparison with healthy controls. However, as soon as this last motor ability is lost, the learning ability is completely lost too (Kübler and Birbaumer, 2008). According to these authors, the minimum capacity to interact with the environment and to be reinforced for successive actions is a necessary prerequisite of intentional learning.

The paralysis argument is also important because it helps to distinguish between phylogenetic, ontogenetic, and actual genetic aspects of consciousness. My main claim that human consciousness emerges in human evolution at an interface between play, tool use, and communication does not imply that the same three components necessarily participate, and in the same constellation, in the individual development of conscious thinking in children. Even less is it to say that the same components necessarily participate in each actual case of conscious thinking. In the development of human behavior, many feedback loops, originally running between the organism and the environment or between the brain and bodily periphery, later on become shorter and remain within the simulating brain (e.g., Grusz, 2004).

To summarize, the objections are not convincing. The former two miss the adaptive function of consciousness and, therefore, presume epiphenomenalism. The paralysis argument requires an additional assumption of similarity between phylo- and ontogenesis and, besides this, ignores the fact that in the rare documented cases of complete LiS (i.e., when no behavior exists), no cognitive function could be found.

### Recursion

The most important implications of the model are recurrent processes at several levels. The best studied class of them is symbolic recursion. When we possess symbols causally free from the elements of the environment they stand for, and arbitrary rules which govern the use of these symbols, we can build symbols which stand for symbols, and rules governing other rules, and symbols standing for a system of rules governing symbols, etc., etc. At least primates (e.g., Flack and de Waal, 2007) and possibly dogs (Watanabe and Huber, 2006) are already capable of metacommunication, i.e., to signals indicating how the following signals should be interpreted. According to Chomsky (1968, 1981), symbolic recursion builds the basis for the infinite complexity of human language.

The second class encompasses instrumental recursive processes automatically emerging with the increasing order of tools. They can constitute highly complex loops, e.g., a machine A produces the machine B which produces the machine C which produces screws necessary to produce the machines A, B, and C.

Recursivity of human consciousness allows us to rebuff another objection that is traditionally put forward against all kinds of instrumentalism. According to the argument, we just need not see objects as tools. For example, we consciously perceive a meadow and trees upon it. Although all of this can be used (cows may pasture on the meadow; fruits may rife on the trees), this usefulness is not normally presented in our consciousness while we are looking at this view. Even in the instance of obviously useful objects (e.g., furniture) we usually perceive these objects without being aware of their instrumental value. I just see my sofa without being afforded to sit down on it. Even less am I aware of continuous action preparation in my consciousness. I see this, hear that, like one thing and dislike another one, but I do not plan any actions with them.

This “neutrality illusion” can be easily explained on the basis of the present model. While each tool use diminishes our personal relatedness to objects, closed loops can completely extinguish this relatedness. In the world of recursive relations between tools producing tools for making tools, I do not feel my personal involvement any longer, but remain an external observer, a passive viewer of the complex systemic relationships between the multiple components of my world, rather than a component of this same system.

Perhaps the most interesting kind of recursive processes, in addition to symbolic and instrumental recursion, is the *temporal* recursion. It will be briefly depicted below in section Memory.

## Properties

A theoretical model of consciousness can be evaluated by the easiness with which the known properties can be deduced from it (Seth et al., 2005). These authors proposed a list of “sixteen widely recognized properties of consciousness” including philosophical (e.g., self-attribution), psychological (e.g., facilitated learning), and physiological (e.g., thalamocortical loops) criteria. Some of these criteria correspond to our everyday experience (“consciousness is useful for voluntary decision making”), whereas others (“sensory binding”) are only valid within the framework of a particular hypothesis which is neither empirically supported (Fotheringhame and Young, 1998; Riesenhuber and Poggio, 1999; Vanderwolf, 2000) nor logically consistent (Bennett and Hacker, 2003; van Duijn and Bem, 2005). The list does *not* include intentionality (Brentano, 1982), nor does it warrant that the 16 criteria are mutually independent (e.g., “involvement of the thalamocortical core” and “widespread brain effects”) and non-contradictory (e.g., “fleetingness” and “stability”).

As our present topic is *human* consciousness, this list of properties to test the model should not, on the one hand, include unspecific properties of any kind of conscious experience. On the other hand, we should not consider properties related to specific historical and cultural forms of human consciousness (e.g., satori), and by no way should we regard those questionable “properties” deduced from particular views on human nature, e.g., such property of consciousness as “computational complexity” (as if the WWW were computationally simple).

On the basis of these considerations, and taking into account space limits, I do not claim to check an exhaustive list of criterial properties of human consciousness, but rather, to illustrate the consequences of the hypothesis using a few representative examples: seriality and limited capacity; objectivity; the intimate relation between consciousness and memory; and the sense of conscious agency.

### Seriality

The serial character of human conscious experience is a highly salient and, from the point of view of many neurophysiologists, an almost mysterious feature. While the brain (which is supposed to be the seat of mind) works as a parallel distributed network with virtually unlimited resources, conscious events are consecutive, happen one at a moment, and their momentary capacity is strongly limited. Theories regarding consciousness as a particular case of brain information processing must, therefore, suggest a specific mechanism creating serial processing from parallel. This compromises the aesthetics of the corresponding theories, because in addition to the multiple brain mechanisms generating conscious contents one more mechanism is postulated to make these contents run in a row.

The difficulties disappear, however, we assume that consciousness has been emerged from behavior and is itself a covert behavior. As already said, human consciousness can be afforded only in specific, particularly complex situations. But any kind of complex behavior is a *series* of organism-environment interactions. A cat do not hunt and wash, or eat and play, at the same time. Likewise, we cannot simultaneously turn left and turn right, notwithstanding all parallel distributed processing in our brain.

An example of locomotion, which is largely unconscious, illustrates the limits of parallel behavior. With automatization of a motor skill organisms acquire the ability to perform some motor acts simultaneously. This process plays a particular role in actively communicating animals such as primates. After extensive experience the muscles of face and tongue become independent of peripheral coordinations, and we can walk and talk at the same time. But as soon as the situation gets more complex, this ability to perform two behavioral acts in parallel is lost. It is difficult and even dangerous to actively communicate while descending an unfamiliar stair in complete darkness. Complex behaviors are serial by nature. In those exceptional cases in which they can run in parallel, the states of consciousness can be parallel, too: whales sleep with one half of the body.

A similar idea was suggested by Merker (2007) who related the seriality of conscious behavior to the existence of a “motor bottleneck” in the bridge (*pons cerebri*) in the upper part of brain stem. However distributed are processes in the cortex, in order to reach muscles the cortical activity must pass through the site where all impulses from the forebrain to the motor periphery converge. Locked-in syndrome discussed in the section Objections above is most frequently a result of a stroke in this area. From this point of view, consciousness is serial because it is restricted by the “common final path” to the effectors, and its limited capacity is a function of the limited capacity of motor activity.

The serial character of human consciousness is closely related to another specific feature, the intolerance of contradiction. Parallel distributed processing in brain networks has nothing against contradiction; different neuronal assemblies can simultaneously process different aspects of information, perhaps incompatible with each other. Both Freudian and cognitive unconscious (Shevrin and Dickman, 1980; Kihlstrom, 1987) are highly tolerant against contradiction. This fact strongly contradicts (sorry for word play) to the negative affect we get as soon as we notice a contradiction between two contents of our consciousness. Again, the paradox disappears when we realize that consciousness is not a processing but a behavior. Mostly, ambiguous behavior is either physically impossible (e.g., simultaneous moving in two directions), or maladaptive (e.g., making one step forth and one step back). Why should consciousness be different?

### Objectivity

This term is used in two interrelated meanings. First, it means that we live in the world which appears to contain distinct and relatively stable entities called objects. Second, “objectivity” means a kind of detachment, i.e., freedom from values, needs, passions, and particular interests. To my knowledge these features are either taken for granted (i.e., the world just consists of objects), or attributed solely to the development of language (e.g., Maturana and Varela, 1990; Dennett, 1991; Damasio, 1999). The former view is not tenable: our being in the world is not a cold and impersonal observation. The latter view is partially true. Operations with signs standing for something different increase the distance between us and the world. Symbolic systems are powerful tools we use to deconstruct the complex world into separate things.

However, in order to use words as tools, we first must use tools. Language can support but not create the objective world. Only tools can do this because they are material components of the same world. Tools put themselves between us and our needs projected into the world (see Tools above). They expand the space *relating* the organism *to* its immediate *Lebenswelt* so much that they transform it into the space *separating* the organism *from* its environment. They enforce me to deal with relationships between different elements of the world, and between different features of these elements, rather than to be ever egocentrically busy by the relationships between the world and myself. They decentralize world. More than one million years ago, the early Homo already employed higher-order tools (Ambrose, 2001; Carbonell et al., 2008). Long before Copernicus stopped the sun rotating around the Earth, tool usage stopped the world to rotate around each animal's needs. In the extent the needs retire into the background, so the related emotions. We can now be engaged into the world of entities which do not immediately concern us. We can, within some narrow limits, remain cool and “objective” (Kotchoubey, 2014).

Symbolic games add a new quality to this objectivity. The two sets of recursive loops (the symbolic and the instrumental) mutually interact, further enhancing detachment and disengagement. When the recursivity of tools added with the recursivity of signs conditionally referred to the tools, the distance between the organism and the world becomes a gap. First the relationship “me-world” was replaced, or at least complemented, with the relationships among objects. Then even these latter are substituted by the relationships between arbitrary symbols standing for objects and their relations.

The higher is the order of tool use, the stronger am I bracketed out of the chain of events. The transformation of the fluent energy of the world into the static order of stable objects finally attains the degree at which I am myself (almost) similar to other things. The living human organism, which is primarily a node of struggling, suffering, enjoying, wanting energies, becomes (almost) just another object of cold cognition among many objects. In the course of this decentration an individual can even get a strange idea that his/her own behavior is caused by external objects, like a behavior of a billiard ball is mechanically caused by other balls hitting it!

In our culture, the objectivity of the world is further strengthened and enhanced by the stance of natural science (Galilei, 1982). From this point of view only quantitative relations among elements of the world are “real,” that is, they belong to the world itself. In contrast, qualities, i.e., the effects of these relations on my own organism, are regarded not as “physical facts” anymore, but as “illusions” of my “mind” (Dewey, cf. Hickman, 1998). Thus color, warmth, loudness, all these proofs of my engagement in the world are just “mental events” indirectly referred to some other (real, physical) characteristics such as wavelength, molecular energy, or amplitudes of oscillations. Interpretation of the relationships *between* us and various aspects of our environment in terms of the relationships *among* these aspects became a criterion of scientific truth.

Thus the physiological opposition between the *milieu intériere* and *milieu extériere* (Bernard, 1865) becomes the philosophical opposition between the Subject and the Object. Both are products of using tools, separating the organism from the world.

### Memory

The relationship between embodiment, memory, and consciousness are discussed in a parallel paper (Kotchoubey, in press) and can only briefly be concerned here. Human consciousness defined as a virtual space for covert anticipatory actions implies an ability to deliberately delay reinforcement (“building a bridge over the gap of time”), thus introducing a strong time dimension. It has even been proposed that the freedom to operate in time, i.e., to activate in one's behavior temporary remote events, conditions, and one's own states, is the *specificum humanum*, the main feature distinguishing humans from all other creatures (Bischof-Köhler, 2000; Suddendorf, 2013). The close correspondence between kinds of memory and kinds of consciousness was first demonstrated by Tulving (1985a,b). Also, he showed on the basis of neuropsychological observations that memory is a bi-directional function, i.e., it relates the organism not only with its past but also with its future (Tulving, 1985b, 1987).

In accordance with this idea, the present model of consciousness is hardly compatible with the classical view that memory is about the past. This view is based on the computer metaphor implying strong separation between storing and processing, which does not exist in biological organisms. From an evolutionary viewpoint, memory was selected not to store the past but to use the past in order to optimize the present behavior and to organize future adaptation. “[T]here can be no selective advantage to mentally reconstruct the past unless it matters for the present or future” (Suddendorf and Busby, 2005, p. 111).This is equally true for short-time memory (STM) as an obvious derivate from *working memory*, which is immediate future planning (e.g., Atkinson et al., 1974). Atkinson and Shiffrin (1971) even identified the actual content of consciousness with the content of STM.

As soon as memory is not regarded anymore as a function of saving information, but rather, as that of behavioral adaptation taking into account the organism's past, many phenomena usually viewed as “errors of memory” became normal. When we are prompted to remember something, we build hypotheses, check them up and adjust them according to the actual situation to other components of knowledge (Bartlett, 1967) as well as to social conditions (Loftus, 1997; Gabbert et al., 2003). In other words, our behavior toward the past does not differ from that toward the present or future. Most so-called errors of memory are not malfunctions, they indicate the flexibility and adaptability of our behavior in the time domain (Erdelyi, 2006). Remembering is neither a faithful recapitulation of past events nor a construction of a reality-independent mental world, but interaction and adaptation (Suddendorf and Busby, 2003).

In the VR of human consciousness an overt action with its real consequences is delayed until the virtual action is virtually rewarded or punished. Therefore, the time dimension, which originally was a flow of events, is now split into several axes. First, the flow of behavioral events is held, as long as no events happen. Second, the sequence of events in consciousness creates a new flow of symbolic events: a second axis, along which we can free move in both directions (Bischof-Köhler, 2000; Suddendorf, 2013). The freedom of moving backwards is of vital importance; otherwise, erroneous actions with their negative consequences would be as uncorrectable as they are in real life. Third, although overt behavior is delayed, other processes (physiological activity at the cellular, tissue and organ levels, as well as automatic actions) go on.

The split time makes human consciousness particularly interesting and dramatic. The combination of the resting external time with the free travel in the virtual time provides us with the ability to quickly actualize (in the sense: make actual, efficient in our behavior) any remote or possible consequence. If we only once ask, “when I do this, what after?” nothing (in principle) can prevent us from asking the same question infinitely many times (e.g., “when after X comes Y, what comes after Y? And what is after the event which will happen after Y?”; etc.). This recursive process renders us to know that the day after tomorrow will be followed by the day after the day after tomorrow, and so on up to the day of our death. But, then, what happens after my death? I don't want to say that all humans really ask these endless “what after?” questions. I want to stress, however, that the ability to realize one's whole life and death and to ask what will follow it is not a product of a particular cultural development, but belongs to the most universal properties of human consciousness and immediately results from the basic structure of anticipatory behavior in the virtual space of symbolic games.

### Voluntary action

The question, why complex human (and animal) behavior is necessarily free, has been discussed in many details elsewhere (Kotchoubey, 2010, 2012). In this text, I shall only concern one particular aspect of this general problem, namely the strong *feeling* of agency, of personal control of one's actions. This issue clearly demonstrates the advantage of the present model of human consciousness over the prevailing cognitive models. These latter assume that the brain first has to make representations of outer objects, and then, this cognitive activity is complemented by actions to deal with these objects. Despite a century of serious critique (Dewey, 1896; Järvilehto, 2001b) this notion is still alive and leads us to ask the question of how the brain can know that my movements belong to me. As always, the answer is postulating an additional “module” of attribution of actions to the agent (de Vignemont and Fourneret, 2004). Thus a cat's brain first makes a decision that she will *jump* for a mouse, and then, she needs an additional decision making that *she* (rather than another cat, or a fox, or a raven) will jump for the mouse.

Such problems do not emerge altogether when we remember that the object of adaptive behavioral control are not our motor actions (the output) but a particular state of affairs (the input) (Marken, 1988; Jordan, 1999). Humans think in teleological terms (Csibra and Gergely, 2007) not because such thinking can be useful but because actions cannot be described in terms other than their outcomes (Hommel et al., 2001). Actions are voluntary if the input patterns they generate can be covertly tested within the virtual space of consciousness.

This definition has important corollaries. It does not require that we are aware of any details of the actions we nevertheless perceive as conscious. The logical impossibility of such awareness was demonstrated by Levy (2005). Equally impossible (and in a blatant contradiction with our intuitive feeling) are the demands that voluntary actions should be preceded by feelings like “wish” or “urge,” or must imply a zero effect of the situation on behavior (e.g., Wegner, 2002). No behavior can be carried out without taking some aspects of the environment into account.

The basis of agency is the simple fact that predators, as a rule, do not attack their own body. This is the difference between “the inside” and “the outside” quite similar to the distinction between the own and alien albumins in the immune system. Of course, this fundamental representation of behavioral actions as “mine” need not be conscious, let alone conscious in the sense of the present article. However, as soon as we admit that consciousness develops from behavior, we understand that this simple me/non-me distinction is a precursor of human agency.

**Figure 3:**
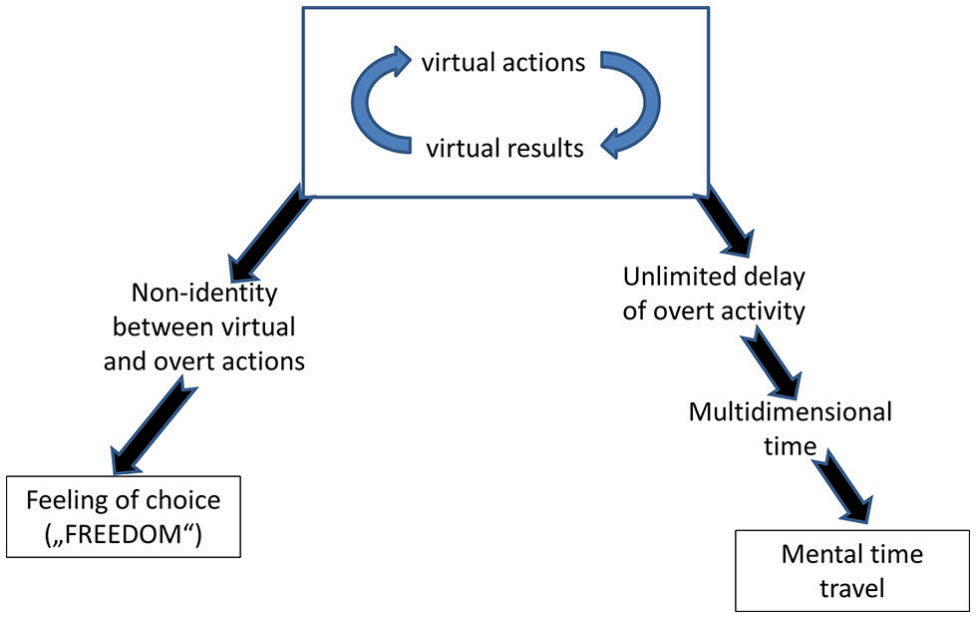
A specific relation of human beings to time and a strong feeling of agency (authorship of one's actions) are regarded by philosophers from Augustinus (2007) to Heidegger (1963) as fundamental features of human consciousness. The scheme shows that these features can be deduced from the model of human consciousness developed in this article.

What makes this agency the fact of our conscious awareness is the choice. Most lay people simply identify freedom with choice (e.g., Monroe and Malle, 2010; Vonasch and Baumeister, 2013; Beran, 2015). Choice is the result of the fact that virtually performed actions can differ from the actions overtly performed. If there is no this difference, i.e., if we always perform the same action that we have thought about, the whole enterprise of “consciousness as VR” would be meaningless. But when this difference exists, it proves that in the same situation at least two different actions were possible, and therefore, we had freedom of choice (Figure 3). In hindsight, we regard an action as voluntary if we did, or could, estimate possible consequences of several alternatives and selected one whose virtual results were the best^1^.

A necessary but not sufficient mechanism of this choice is inhibition of overt behavior. Therefore, the view that associates volition with the ability to exert inhibitory control of otherwise involuntary actions (veto: Libet, 1985) deserves attention. Human conscious activity strongly correlates with activation of those brain structures whose main function is inhibition. These structures are specifically active during particularly complex forms of human behavior. However, inhibitory control is a precondition of volition but not volition itself. If I have to repair my car, I must stop it first; but stopping is not repair. The decisive point is not veto but choice.

## Other views

A good theory does not only shed light at its object, but also at the other views on the same object. As a famous example, the relativity theory not just explains the mechanisms of the Universe; it is also successful in the explanation of why other respectable theories (e.g., Newtonian mechanics) gave a different explanation of the same mechanisms: because they regarded a very limited range of possible velocities. Likewise, from the point of view presented here the origins of several alternative theories of consciousness can be apprehended. Of course, this highly interesting task cannot be pursued in full in this paper; we cannot discuss all existent theories of consciousness in their relationship with the current model. Rather, I shall restrict the review to the approaches apparently similar to the present one.

### Embodiment theories

The proposed theory is most similar to embodiment theories of consciousness, simply because it is one of them. Embodiment theories are characterized by “4Es”: human experience is *embodied* (i.e., brain activity can realize mental processes only being involved in closed loops with the peripheral nervous system and the whole bodily periphery including inner organs and the motor apparatus), *embedded* (i.e., the brain-body system is further involved in closed loops with the environment), *enacted* (i.e., in the interaction with the environment, brain and mind not just process information but make the organism to play the active role *pursuing its goals* notwithstanding environmental disturbances), and *extended* (i.e., it involves external devices as if they were parts of the same brain-body system) (e.g., Tschacher and Bergomi, 2011).

Beyond the general agreement at these four points, different embodiment theories of mind and consciousness build a very broad spectrum varying in their account on the exact role and mechanisms of realization of each point, as well as interactions between them. The hard discussions running in the last decades within the embodiment camp would, however, lead us far beyond our present topic; they are addressed, e.g., in the publications of Menary (2010), Bickhard (2016), Stapleton (2016), Tewes (2016), and the literature cited there.

To be sure, the present approach shares these four E-points. Particularly, anticipatory regulations we have begun with, are closely related to the principles of embeddedness and enactiveness; and the critical role of tools in my approach fully corresponds to the principle of extendedness.

However, to my best knowledge no embodiment theory has up to date been devoted to the issue of the origin and the biological basis of specifically human awareness. Rather, several representatives of this approach attacked the hard problem of the origin of elementary forms of sentience or perceptual experience (e.g., Varela et al., 1991; O'Regan and Noe, 2001; Jordan, 2003; Bickhard, 2005; Noe, 2005; Jordan and Ghin, 2006). How successful these attacks have been, should be discussed elsewhere. From my point of view, the sensorimotor theory (Hurley, 1998; Noe, 2005) has not convincingly responded to arguments raised by it critics (e.g., Hardcastle, 2001; Kurthen, 2001; Oberauer, 2001) who indicated that even a best explanation of mechanisms and phenomena of perception does not imply an explanation of perceptual experience, that is, “what it is like” to perceive a red color or a high-pitch tone. If we assume that simple robots do not have conscious experience, the fact that the proposed embodied and enacted mechanisms of perception can be modeled in robots already refutes the idea that these mechanisms can explain consciousness.

The sensorimotor theory is, of course, only one of the embodiment-grounded attempts to explain the emergence of consciousness. Other (“interactivist”: Bickhard, 2005, 2016); or (“relational”: Jordan and Ghin, 2006) approaches, having a more profound evolutionary foundation, may be more successful in this enterprise. Nevertheless, they have not yet given any systematic account of the transition from the alleged simple sentience to human consciousness, which is the theme of the present paper.

### Simulation theories

Part 2 above exposed the idea that human consciousness is a secure space where behavioral actions are virtually performed, and their consequences are virtually apprehended. In general, this idea is not new but goes back to the British associationism of the eighteenth century (Hesslow, 2002). In experimental psychology, the concept of cognition as “covert trials” was advanced by Tolman (e.g., Tolman and Gleitman, 1949; Tolman and Postman, 1954) and in philosophy, as the theory of conjectures and refutations (Popper, 1963). It is further in line with the well-known scheme of “test-operation-test-exit” (Miller et al., 1960). About 40 years ago, Ingvar (1979; also Ingvar and Philipsson, 1977) practically formulated the concept of consciousness as anticipatory simulations; unfortunately, he justified his conclusions by brain imaging data which appear to be of questionable quality today, not surprising given the enormous progress of brain imaging techniques since then.

The same idea of covert behavior underlies the concept of efference copy (von Holst and Mittelstaedt, 1950), as well as some control-theoretical models that regard themselves as alternatives to the efference copy theory (e.g., Hershberger, 1998). In the last decades similar views were thoroughly developed under the terms “simulation” (Hesslow, 2002, 2012) and “emulation” (Grusz, 2004). Particularly interesting from the present viewpoint are the data that virtual performance of actions includes anticipation of action results with simultaneous inhibition of the overt execution of these actions (Hesslow, 2012). Behavior, originally realized in large feedforward loops including bodily periphery and the environment, can subsequently be reduced to the loops within the brain.

Notwithstanding the clear similarity between my VR metaphor and all these old and recent views, there are substantial differences as well. Thus the notion of motor simulation frequently defines “behavior” as purely motor activity separated from perception and anticipation of results. The present approach is, in contrast, based on the presumption of the control theory that behavior is *control of input* rather than control of output and cannot, therefore, be regarded as a set of commands sent to muscles. The very sense of a virtual behavior is obtaining its virtual consequences. A related minor point is the idea shared by many adepts of simulated behavior that a motor system has some “point of no return,” so that when a simulated motor activity attains this point, the movement cannot be inhibited anymore and must be executed. The concept of the “point of no return” is a leftover of motor control ideas of the nineteenth century and has no place in the modern movement science (e.g., Latash, 2008, 2012).

But notwithstanding these rather minor differences between all these approaches (regarded above in a cavalry manner) and the present one, there is a very big difference in the *kind of explanation*. The primary interest of simulation theorists is a *how*-explanations. They ask, how, i.e., using what kind of mechanisms, virtual behavior is realized. My point, to the contrary, is a *why*-explanation: why virtual behavior is realized thus and not differently. For example, without the phylogenetic roots in playing behavior, simulated activity could not possess its astonishing freedom to initiate any virtual action in any circumstances, to interrupt or terminate at any deliberate point and to re-start at any moment. The components of communication and tool usage also have profound effects on the nature of human consciousness, as we shall see in the next session.

### Language and thought

The matter of this section is not properly a theory or a class of theories but rather a group of loosely related views, converging on a literal understanding of “con-sciousness” as “shared knowledge” (lat con-scientia). Thus consciousness is regarded as the product of cognitive activity converted into a form of language to be shared with others. The idea that, roughly speaking, “human consciousness is animal consciousness multiplied by language” has, in fact, become a matter of general consensus as a component of almost all theories of consciousness, even between the authors so different as Edelman (1989), Dennett (1991), Maturana (1998), Järvilehto (2001a,b), and Humphrey (2006), who barely agree at any other point. Indeed, what else is specific for human (in contrast to the animal) consciousness if not the fact that it is based on social cooperation and language-mediated communication?

Crudely, many socio-linguistic theories may be classified into pre-structuralist (e.g., Vygotsky), structuralist (e.g., Levi-Strauss) and post-structuralist (e.g., Derrida). The first stress the process of internalization in which social (interpersonal) processes are transformed into internal cognitive (intrapersonal) processes. Consciousness, from this point of view, is the pattern of social relations (for example, a child-parent interaction) transported into the head. The second class of theories contends that consciousness is based upon hidden cultural and linguistic stereotypes (e.g., the opposition “cultural” vs. “natural”) creating stable, largely a-historic structures. The third view insists on the virtually absolute relativity of the structure and content of conscious human behavior and (in contrast to structuralism) its historical and ideological interpenetration.

Above, when discussing the interaction of communication and play, we already mentioned that human consciousness is frequently regarded as a symbolic game, and that this view is usually traced back to Wittgenstein: “*The limits of my language* mean the limits of my world” (Wittgenstein, 1963; Section 5.6, emphasis in original).

This view, however, leaves unclear wherefrom the structures (or the rules of the game) take their stability and causal power if they are not filled by the content of a language-independent world. Post-structuralists capitalized on this inconsequence and proposed a radical solution for the above problem: if consciousness does not have any meaningful content besides the rules and structures of the game, then, it does not have any rules and structures either (Derrida, 1975). Thus even the notion of symbolic game became much too restrictive since it may imply that there is something the symbols stand for—but in fact, they stand for nothing. Any kind of human behavior is just a “text,” which can be interpreted in a variety of possible ways. For itself (i.e., before and beyond this process of interpretation) there is no such thing as the meaning of an action. Also the world, the so-called “nature” or “reality,” is a text to be interpreted and deconstructed (Foucault, 1966). Not only, therefore, everything is merely a sequence of signs, but these signs do not signify anything: the classical opposition between the signifying and the signified (de Saussure, 1983) is thus annulled. Hence, consciousness is not a game, as previous socio-linguistic theories regarded it, but rather a free play (Derrida, 1975) whose rules may appear and disappear like clouds in a windy day. From the early socio-linguistic point of view, consciousness is its own manifestation in systems of signs. From the later socio-linguistic point of view, consciousness is just these systems of signs and nothing more. “Cognition is a relationship of language to language” (Foucault, 1966; Ch. 1.4).

One can say that these views evolved from the theories of socio-linguistic *foundation* of consciousness, peaking in the linguistic *determinism* in Wittgenstein (1963) and Whorf (1962), to the theories of the unlimited *freedom* of consciousness in its historic and linguistic realization. This freedom, from their (and my) point of view, largely roots in the freedom of the sign, which, in its development from index to symbol, abandoned its causal link to its reference. Importantly, the notion of language as a symbolic game is not limited by syntax. Rather, it is the very meaning of the words which is determined by their location within the network of tacit verbal rules. E.g., we cannot understand the meaning of the word “hard” without its oppositions such as “soft” or “easy.” Also the meaning of mental concepts is nothing but their usage in language, i.e., their position in the linguistic game. Understanding consciousness means understanding how the term “consciousness” is used in our culture (Bennett and Hacker, 2003).

Because many very influential linguistic theories originally accrued in philology and cultural anthropology, they may appear to concern only particular forms of consciousness expressed, e.g., in arts and letters but not the basics of human consciousness. This is not true. These ideas profoundly affected the contemporary thinking on mind and consciousness down to the level of such “elementary” functions as visual perception (e.g., Gregory, 1988, 1997) and neural development (Mareshal et al., 2007). They left their trace even in strongly biological approaches to cognition and consciousness (e.g., Varela et al., 1991; Maturana, 1998).

From the present point of view, socio-linguistic theories correctly emphasize communication and play as important sources of human consciousness. Most elaborated of them also stress its prospective nature making conscious behavior “free” in the sense of being not determined by the past. However, all these views, traditional and contemporary, philosophically or biologically oriented, completely miss the instrumental nature of human behavior. Many of them talk about tools; e.g., they regard words as tools, scientific theories as tools, etc. But besides this, our consciousness is based on simply tools, which are not words, not theories, just tools. Using them, we either attain our goal (if we correctly grasp the objective relation between elements of the environment and their properties), or not (if our conceptions are wrong). Thus the results of tool usage continuously test the validity of our symbolic games. “By their fruit you will recognize them”(Bible: Mt. 7, 16). This fruit is the banana, which Köhler's (1926) apes reached using sticks and boxes. If their concepts of sticks and boxes were true, they reached the banana, but when they were false, they remained hungry.

It is true that, e.g., a building can be regarded as a “text,” and that the architect may have projected his personality into his drawings. But in addition, the building has to withstand gravity, wind and possibly earthquakes. To understand the meaning of “hardness,” it is important to recognize its relationships within the field of its use in the language (e.g., the hardware/software distinction). But it is also important to remember that our ancestors failed to reach bananas using a bundle of straw, simply because the bundle was not hard.

### Common working space

The theory of common working space (CWS: Baars, 1988, 1997) is probably the most elaborated *psychological* theory of consciousness in the last 30 years. The theory regards the mind as a coordinated activity of numerous highly specialized cognitive modules (Fodor, 1981, 1983) whose work is largely automatic. When some of these specialists meet a processing task for which no preprogrammed solution is available, they build a CWS to make this task as well as all proposed solutions open for every other module. This can be compared with a large audience in which many small groups work each with its own problem, but there is also a possibility to broadcast a problem for the whole audience. Consciousness is this broadcasting; there is a competition for access to it, because the space is only one, and the tasks are many. Therefore, the most interesting processes determining the content of our consciousness are not those which happen in consciousness but those which decide what specialized module(s) should get access to it.

The CWS theory not only provides an explanation for very many characteristic properties of consciousness, but it is also quite compatible with other interesting theories (e.g., reentrance theory), which we cannot discuss due to space limitation.

The metaphor of consciousness behind the CWS model is that of a theater (Baars, 1997). The CWS can be regarded as an open scene accessible for all cognitive modules. The similarity between the theater metaphor and the VR metaphor is obvious. Both presume a scenery, a show, thus pointing to one of the key components of the present hypothesis, i.e., *play*. Both theater and VR are spaces where things are played.

But in this play, we should not play down the differences between the two metaphors. A theater presumes many spectators, who rather *passively observe* the actors' activity, whereas a VR is concentrated around a single participant, who is *actively engaged* in this reality. Furthermore, arbitrariness is much stronger in the theater than in the VR. Millions of people admire opera theater in which they witness how personages express their emotions by continuous singing, which would appear strange and silly in real life.

Also interestingly, the theater metaphor does not warrant the uniqueness of consciousness. Many cities have several theaters, and some people can visit two or three on an evening. Nevertheless, the most established version of the CWS theory assumed that there exists only one common space for each brain (and each body, Shanahan, 2005). Many concrete predictions of the CWS theory result from the assumption of the strong *competition* between modules striving for the access to the only possibility to broadcast. Later on Baars (2003) suggested that there can be multiple CWSs working in parallel. This raises questions such as: what can count for a space to be regarded as “common,” and how many specialized processors (may be only two?) should be connected to build a “partial consciousness.”

It cannot be denied that we normally experience one particular state of consciousness each moment, in accord with the old philosophical idea of the “unity of consciousness” (James, 1890). Baars (1997) and Dennett (1991) devoted a lot of intriguing pages to the issue of how this unity can be created by the distributed brain. Neuroscientists (Singer, 1999; Treisman, 1999; Tallon-Baudry, 2004) regard this question as the main question of the neurophysiological underpinnings of consciousness.

Thus we are surprised that we have only one state of consciousness at one time, despite millions of parallel functioning neuronal circuits in our brain. However, we are not surprised when a big animal (e.g., a whale) makes a jump as a whole, although its body consists of many thousands simultaneously (and to a large extent, independently) working cells. We don't regard this unity as a miracle and don't postulate a specific mechanism of binding these cells into a single organism.

Complex behavior is realized in the form of muscular synergies (Bernstein, 1967; Gelfand et al., 1971; Turvey, 1990; Latash, 2008), which dominate the actual distribution of muscle forces each moment of time. These synergies are motor equivalents of the CWS. The unity of consciousness is the unity of behavior. This does not mean that the unity is unproblematic, but the analogy with motor control indicates the correct name for the problem. The motor system does not have a binding problem but must solve the problem of excessive degrees of freedom, also called “Bernstein problem” (Bernstein, 1967; Requin et al., 1984; Latash, 2012). The principle of “freezing degrees of freedom” implies that muscles are not permitted to work independently, but all must remain within a frame of a unifying synergy. With the development of a motor skill, the synergy becomes more and more local until it is limited to those muscles only, whose participation is indispensable.

Of course, when we talk about muscles we also mean the whole nervous apparatus these muscles are connected with. Therefore, as far as the unity of the CWS is the unity of complex behavior, there is no contradiction between the CWS theory and the present one. Accordingly, the control of new, unskilled actions is frequently conscious. The question is *why* the common working place of consciousness is common. From my point of view, it is not because a group of processing modules has decided, in a democratic or dictatorial way, that a given piece of information is interesting enough to make it accessible for the whole audience, but because complex behavior cannot be organized other than by coordinating all activity to a common pattern. Likewise, we do not make two conscious decisions simultaneously not because the two must compete for one scene, but because, if we did make them simultaneously, how would we realize these decisions? The answer is: serially, one after the other.

## Conclusion

The model is presented that conceives of human consciousness as a product of a phylogenetic interaction of three particular forms of animal behavior: play, tool use, and communication. When the three components meet in humans, they strengthen and mutually reinforce each other producing positive feedback loop. Therefore, although all three elements of human consciousness are present in many animal species (not necessarily human predecessors), there is no other species that plays, communicates and uses tools as much as humans do.

The suggested three-component structure permits to easily explain most typical features of human conscious awareness: its recursive character, seriality, objectivity, close relation to semantic and episodic memory, etc. Other specific features of human consciousness (e.g., the emotion of anxiety) remain, unfortunately, not discussed due to space limits. Finally, a comparison of the current approach with other theories of consciousness (embodiment theories, simulation theories, common working place) reveals, notwithstanding some similarities, important differences from all of them. Again due to space limits, the complex relationships of this model of consciousness with the multiple draft theory, the re-entrance theory, and the classical dualistic approach must remain outside the present text.

## Author contributions

The author confirms being the sole contributor of this work and approved it for publication.

### Conflict of interest statement

The author declares that the research was conducted in the absence of any commercial or financial relationships that could be construed as a potential conflict of interest.

## Footnotes

^1^An in-depth discussion about the meaninglessness of the question, whether a different action could be chosen in the case of the exact repetition of the same situation, is given in Kotchoubey (2012, 2017). To put it briefly, meaningless is primarily the term “exact repetition.”
